# Life strategy of Antarctic silverfish promote large carbon export in Terra Nova Bay, Ross Sea

**DOI:** 10.1038/s42003-024-06122-8

**Published:** 2024-04-11

**Authors:** Clara Manno, Erica Carlig, Pier Paolo Falco, Pasquale Castagno, Giorgio Budillon

**Affiliations:** 1 British Antarctic Survey, Natural Environment Research Council, NERC, Cambridge, UK; 2 National Research Council (CNR) of Italy, Institute for the study of the Anthropic impacts and the Sustainability of the marine environment (IAS), Genoa, Italy; 3 Department of Life and Environmental Sciences, Marche Polytechnic University, Ancona, Italy; 4 Department of Mathematics and Computer Sciences, Physical Sciences and Earth Sciences (MIFT), University of Messina, Messina, Italy; 5 Department of Science and Technology, University of Naples “Parthenope”, Naples, Italy

**Keywords:** Biological sciences, Biochemistry, Climate-change ecology

## Abstract

Antarctic silverfish *Pleuragramma antarcticum* is the most abundant pelagic fish in the High Antarctic shelf waters of the Southern Ocean, where it plays a pivotal role in the trophic web as the major link between lower and higher trophic levels. Despite the ecological importance of this species, knowledge about its role in the biogeochemical cycle is poor. We determine the seasonal contribution of Antarctic silverfish to carbon flux in terms of faeces and eggs, from samples collected in the Ross Sea. We find that eggs and faeces production generate a flux accounting for 41% of annual POC flux and that the variability of this flux is modulated by spawning strategy. This study shows the important role of this organism as a vector for carbon flux. Since Antarctic silverfish are strongly dependent on sea-ice, they might be especially sensitive to climatic changes. Our results suggest that a potential decrease in the biomass of this organism is likely to impact marine biogeochemical cycles, and this should be factored in when assessing Southern Ocean carbon budget.

## Abstract (web summary)

A study focused on the Ross Sea found that sinking of Antarctic silverfish eggs and faeces promotes the biological carbon pump contributing to a large amount of the annual carbon export.

## Introduction

The world’s oceans are estimated to absorb about 25% of the total CO_2_ emissions from the atmosphere every year^1^, contributing to climate mitigation. This process is supported by the Biological Carbon Pump, the fixation of inorganic carbon (C) through photosynthesis by phytoplankton, and subsequent C export and sequestration to deeper waters. Recent interest in the role of fish in biogeochemical cycles has highlighted the need for better parameterisations of C export generated by these organisms^2^.

Despite the global ecological and economic significance of fishes, observations, and process studies of their contributions to the ocean carbon export are poor^3,4^. However, due to the high abundance of epipelagic fishes on coastal shelves and in upwelling regions, and the high biomass of mesopelagic fishes in the vast expanse of oligotrophic regions, their contribution is likely to be important and has recently estimated as 16% of total carbon flux^5^.

The Southern Ocean plays a pivotal role in the global carbon cycle and in the global C uptake^6–8^. Rapidly sinking faecal pellets generated from zooplankton are a major contributor to the biological pump and the export of carbon^9–12^. However, studies from other regions show that sinking of large faecal pellets produced by fish can reach over thousands of metres per day^13,14^ which is generally one-two order of magnitude higher of the sinking speed of zooplankton faeces. Moreover, the cohesive nature of fish faecal pellets suggests that they are resistant to bacterial decomposition which combine with high sinking rates facilitate their descent to the benthos^14^.

The Antarctic silverfish *Pleuragramma antarcticum* is a nototheniid species widely distributed in waters of the continental shelf in High-Antarctic regions. It is abundant, accounting for about 98% by number of the total ichthyoplankton on Weddell Sea continental shelf and accounts for over 90% of the pelagic fish biomass in the coastal area of the Ross Sea^15–18^. *P. antarcticum* is a key species in the marine trophic web, since it plays a pivotal role in the trophic structure of the High-Antarctic coastal ecosystems by channelling energy flow between plankton and the top predator community^19–21^. It feeds largely on zooplankton, but has shown a flexible and opportunistic trophic strategy, relying on the wide variety of planktonic prey^22^. As a prey, P. antarctica provides a high energetic value^23^ and occurs in the stomach of predators ranging from large fishes, like the Antarctic toothfish, to penguins, marine flying birds, and seals^21,24^. Among notothenioids, *P. antarcticum* is the only known holopelagic species, living all stages of its development within the water column, exhibiting a complex lifestyle: eggs develop under fast ice, larvae and juveniles disperse over the continental shelves and adults live in the coastal areas around the Antarctic continent^17,18^. Given its abundance and ecologic role, the Antarctic silverfish has been extensively studied on many aspects of its biology and ecology^17,18^. Nevertheless, quantitative knowledge on the role this organism plays in the biogeochemical cycle is absent.

Here, we determine the seasonal contribution of Antarctic silverfish to the flux of carbon as faeces and eggs from samples collected by a moored sediment trap deployed for one year on inshore area of Terra Nova Bay, the Ross Sea, in the Western sector of the Southern Ocean. This region is considered the only nursery and hatching area of the Antarctic silverfish where huge quantities of embryonated eggs can be detected floating in huge amounts among the subsurface platelet ice layer under sea ice^18,25^. Platelet ice consists of various-sized flat plate-like crystals up to above 10 cm in diameter, randomly oriented, mostly occurring under the coastal cover^26,27^. Platelet ice is found as a component of fast-ice cores, at depths larger than 1 m^28^, making up a semi-consolidated layer ranging from a few centimetres to metres in thickness^29^. Due to its prominent occurrence under the fast ice, platelet ice is considered a coastline high-latitude feature, although its real extent is still unknown^20^.

The surface ice platelet layer is highly porous, which facilitates nutrient exchange with seawater and supports exceptionally high algal biomass^29,30^. The quantifying of energetic fluxes among the biota, pack ice, platelet layer, and free sea ice water are of scientific interest for understanding the high Antarctic carbon cycle^31^.

This study provides new insights on the importance of the Antarctic silverfish as a vector for C export in a region, such as the Ross Sea, that contributes significantly to global atmospheric C uptake^32^. We found that both eggs and faeces can significantly contribute to the carbon export in this region. As during the entire life cycle the Antarctic silverfish appear to be strongly dependent on sea ice, this species would be especially sensitive to climatic or oceanic changes that reduce the extent and alter the dynamic of sea-ice cover^17,20^. To increase confidence in forecasting the effects of climate change on the biogeochemical cycle, the influence of a changing climate on Antarctic fish must be taken into account when assessing Southern Ocean carbon budget.

## Results

### Eggs and faeces contribution to the POC flux

**Fig. 1:**
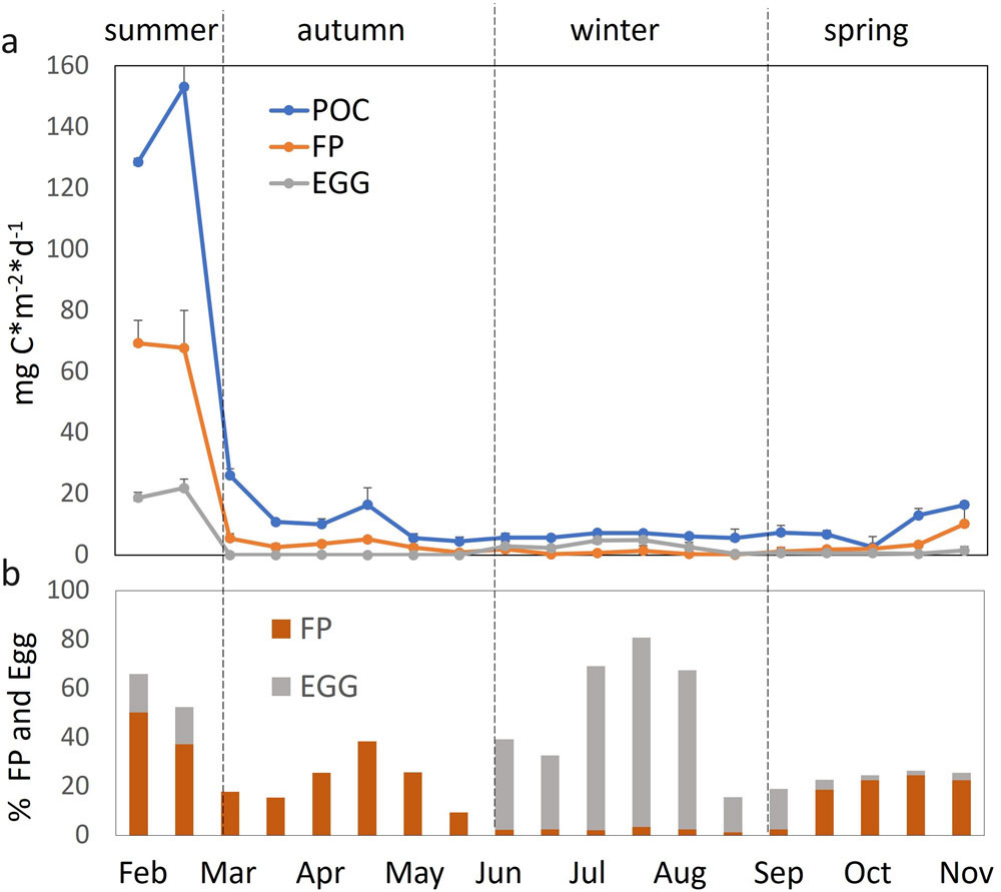
Seasonal trend of carbon flux and Antarctic silverfish Faecal Pellets (FPs) and Eggs contribution. **a** Particulate Organic Carbon (POC) flux (mg m^−2^ d^−1^; ±1 SD) and flux of fish faecal pellets (FP), and eggs (mg m^−2^ d^−1^; ±1 SD). Error bars are standard deviations from replicates (sub-samples) from each cup. **b** relative contribution of fish FP and eggs to total POC (%, ±1 SD).

The trend of Particulate Organic Carbon (POC) flux showed a strong seasonal variability, with values ranging between 5.61 and 153.21 mg C *m^−2^ *d^−1^ (Fig. 1a). POC flux is at a maximum in the summer (February), after which there is a decrease to a low winter level (June to September) until it increases again in the late spring (October onwards).

The relative contribution of the different fish components to total POC flux varies between seasons (Fig. 1b). Eggs and FPs both strongly contributed to the POC fluxes in the summer (accounting together for up to 65.3%) while in the autumn and spring POC contribution is largely characterised only by FPs. Conversely, eggs become the dominant contributor in the winter periods (up to 77.2% of total POC flux) (see Supplementary Table 1 for POC, FP and Egg flux data). Out of an annual total POC flux FPs make the highest contribution (25.3%), compared to eggs (15.2%).

FP and egg carbon content significant varies between the seasons (FP: *z* = −2.8, *p* < 0.05; Egg: *z* = −2.1, *p* < 0.05). In general, FP C values range between 10.9–22.5 µg C mm^−3^ and are at their lowest during the autumn–winter period and highest during spring-summer. Conversely, the carbon contents of eggs present highest values during the winter (55.6 µg C egg^−1^) and lowest during the summer (45.4 µg C egg^−1^) (Table 1).

**Table 1:** Season Variability of fish Faecal Pellets (FPs) and Egg carbon content

	FP-C	SD	EGG-C	SD
Spring	20.51	5.23	51.75	4.56
Summer	22.56	4.34	45.43	2.52
Autumn	12.31	4.65	NA	NA
Winter	10.94	3.12	55.67	4.34

FPs and Eggs carbon content is expressed as µg C mm^−3^ and µg C egg^−1^ respectively.

*SD* standard deviation.

### Seasonal variability of eggs and faeces size

**Fig. 2:**
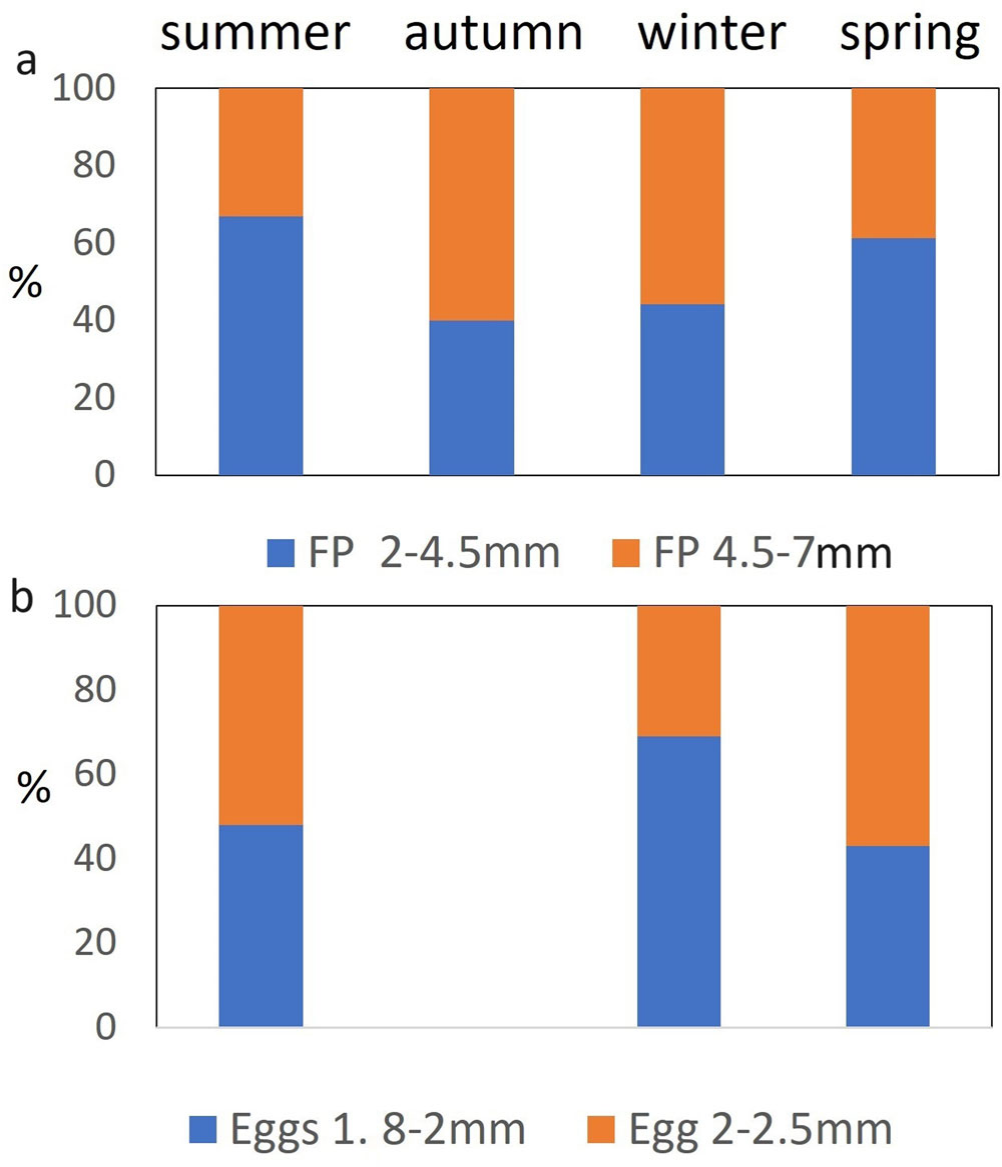
Seasonal variability in the class size of Antarctic silverfish Faecal Pellets (FPs) and eggs. **a** Faecal Pellets (FPs) class size (expressed as %) and **b** Eggs class size (expressed as %). Note most of the fish FP are present as fragments.

All the fish faeces collected in the sediment trap present a light brownish colour and are shaped like long cylindrical, most of them are in the form of fragment with different length ranging from 2.3 to 7.5 mm and were similar in width (0.9–1.3 mm) (Fig. 2a). There was a significant amount of small FP fragments with small size (>60%, up 4.5 mm) in the spring/summer than in autumn/winter (<40%, up 7 mm) (*z* = −2.8, *p* < 0.05). Eggs present a spherical shape with a size between 1.8–2.5 mm (Fig. 2b). Eggs present a significant seasonal variability with a large diameter in the summer and spring (>54%, up 2.5 mm) than in the winter (*z* = *−2.9*, *p* < 0.05) (see Supplementary Table 2 for fish FP and egg class size data).

### Preservation state of eggs

**Fig. 3:**
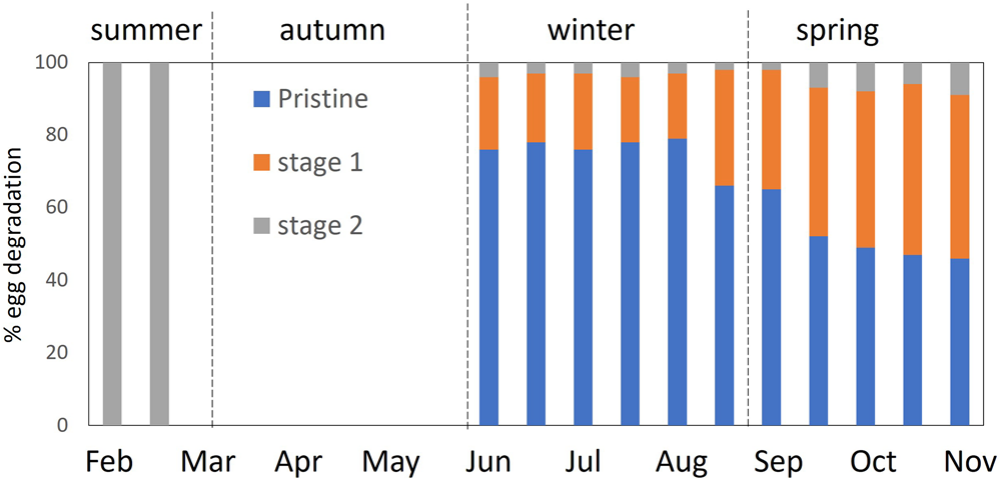
Seasonal variability in the state of egg degradation of Antarctic silverfish collected in the sediment trap. State of degradation of eggs over the year (expressed as %). Pristine (no sign of degradation), stage 1 (early stage of decomposition), stage 2 (advanced stage of decomposition, i.e. physical decay).

During the winter to the beginning of the spring period most of the eggs (up 80%) present a good preservation state with no sign of decomposition, conversely in the summer a high percentage (100%) of eggs revel clear sign of degradation with the presence of physical decay (Fig. 3). In particular, in the most of the eggs present chorion damage (see Supplementary Table 3 for fish egg degradation data).

## Discussion

We found that Antarctic silverfish can strongly contribute to the POC flux in the region of Terra Nova Bay (TNB), in the Western Ross Sea. Their contribution is mainly generated from the faecal pellets and eggs, which together, comprised 41% of the annual POC export in this study region. Hence, we suggest that the unique features of Antarctic silverfish promote the strongest carbon sink in the Southern Ocean regions where this organism spawn (such as TNB).

The potential important role of fish in promoting C flux has already been highlighted by ref. ^5^, who estimated based on a synthesis of passive (faecal pellet sinking) and active (migratory) flux of fishes, a contribution of about 16% to total global carbon flux out of the euphotic zone. To the best of our knowledge this is the only study focuses on the role that fish FPs are playing in the Southern Ocean C export. Further, no previous studies (in the Southern Ocean or elsewhere) have investigated the impact of eggs.

Here we show that eggs can provide 15% contribution to the annual POC, indicating that estimation of global fish input to the carbon cycle need also consider this component. In addition, the relatively large contributions of Antarctic silverfish eggs to the POC in the winter (>60%), promote a relative high pulse of carbon flux in this season (always >5 mg/m^2^/d) which is comparable to POC flux value observed during the productive period (i.e. spring-summer) in the Ross Sea region^33–35^. Therefore, the winter eggs flux may be an important source of nutrition to fuel the benthos.

The highest egg POC flux is observed in the summer (21 mg*m^−2^*d^−1^). Eggs size is significant higher during this period compared to winter, showing an advanced stage of embryo development. Previous studies have suggested that spawning occurred in the winter (July–August) and hatching in spring-summer^25,36^. We suggest that eggs collected in the sediment trap in this period (summer) died before reaching the hatching phase and then start to sink. In fact, most of the sunk eggs (85%) in the summer show evidence of chorion damage and all of them show an advanced state of degradation. Further, incubation experiments show that eggs with an intact chorion could survive and remain viable at temperatures as low as −9 °C but once the chorion is breached by ice, freezing is instantaneous^37^. Pelagic eggs owe their buoyancy almost exclusively (90%) to the high-water content, whereas lipid accounts for only about 10%^38^. Thus, chorion damage can result in a change of the water-lipid balance and lead to the loss of the eggs’ neutral balance. Conversely, in the winter, eggs are pristine and smaller, suggesting they start to sink soon after spawning takes place. Part of the egg mass that sink in the winter might be a result of incomplete fertilisation, despite we cannot exclude that fully fertilised eggs just die relatively soon after they are released. Other mechanisms could impact the ability of fish eggs to maintain neutral buoyancy. Some experiment shows that the quality of fish eggs decreases with batch number^39^, which in turn could decrease the chance of eggs survival and increase the number of sinking eggs^40^. Further, we find that carbon specific content in eggs is higher in the winter (55.6 µg*egg ^−1^) and lower (45.4 µg*egg^−1^) in the summer. The seasonal variability in *P. antarcticum* eggs carbon content agrees with previous experiments carried out on eggs of *Gadus morhua*^41^. The authors show that C content of incubated eggs decrease by about 14% from the newly fertilised eggs until just before hatching since the yolk sac is used to fuel energy for the forming larvae. The egg size after fertilisation of the Antarctic silverfish reported in literature (1.8–2.0 mm)^42^ is the same range of the eggs size observed in the winter. Together, both features (relatively high C content and size range) of the eggs collected during the winter suggest that these are most likely dead-fertilised eggs. Eggs are almost absent in the trap during autumn. This could be explained by adult Antarctic silverfish behaviour which are selecting their spawning habitat during autumn when sea ice is also beginning to advance^43^.

Maximum flux of FP-POC (69 mg/m^2^/d) is one order of magnitude lower comparing to maximum value observed in previous studies carried out on Northern anchovy (i.e. 251 mg C m^−2^ d^−1^)^14^ which can be related to the behaviour of this fish to school and then aggregate in large number. Conversely Antarctic silverfish exist as loose shoals^44^ (unstructured aggregations), with individuals estimated to be spaced 2–4 m apart at densities of one fish per 7–43 m^3^, despite in the Weddell Sea, it have been occasionally observed in dense aggregations^45^. We observed a significant decrease in FPs fragment size in spring and summer. This may be, in part, a result of the highest biological activities (i.e. presence of zooplankton) in the water column during this period which could increase the FPs fragmentation and in turn remineralization process. This is also supported by the presence of large number of zooplankton FPs observed in the same samples during spring-summer (Manno *personal observation*). Conversely the large size fragment (but low number) of FPs observed in winter reflect the decrease of physiological activity of fish during spawning, resulting in a seasonal reduction in feeding because most of the energy is devoted to mature eggs. Moreover, in the winter a low productivity water column allows fish FPs more likely to sink without encountering too many biological barriers. The continuous presence of Antarctic silverfish FPs over the sampled period in the sediment trap is an agreement with the ecology of this organism which, despite being a pelagic fish, is mainly a relatively inactive species and does not form dense continuously swimming schools^17^. FPs contains almost the double amount of carbon in the spring/summer compared to winter, reflecting the variability in food availability and quality throughout the seasons^9^ find a similar trend in the Scotia Sea (Atlantic Sector of the Southern Ocean) where FPs produced by zooplankton present twice the amount of carbon in the productive season than in the winter.

**Fig. 4:**
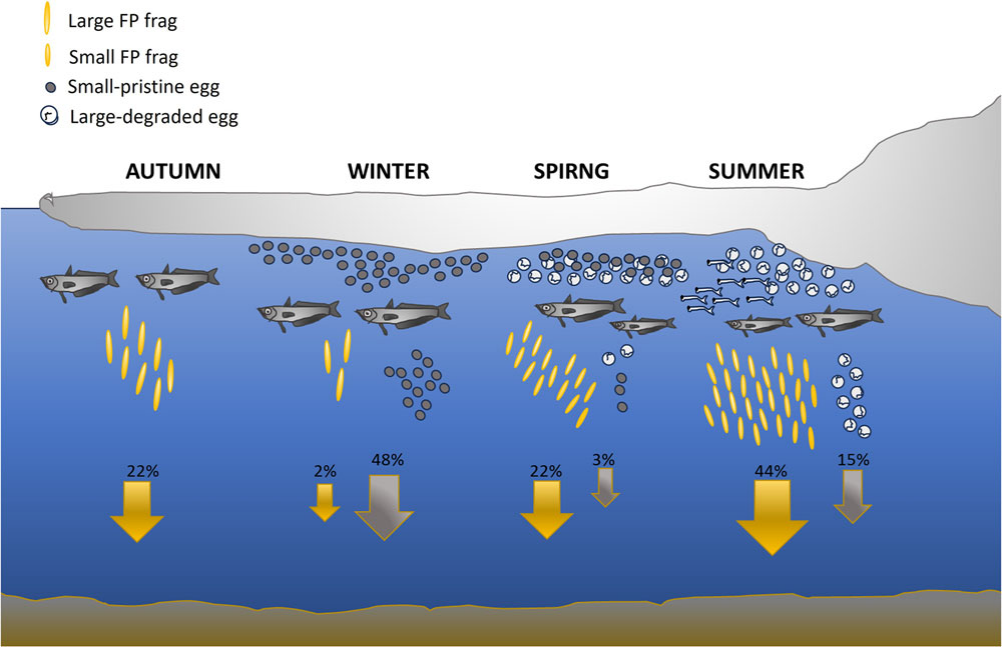
Schematic representing the seasonal contribution of Antarctic silverfish eggs and Faecal Pellets (FPs) to the Particulate Organic Carbon (POC) in Terra Nova Bay. The contribution of FP and eggs to the seasonal POC is expressed as % and intensity is indicated by the size of yellow (FPs) and grey (eggs) arrows. The schematic highlights the variability in FP and Egg class size between the season as well as the change in egg degradation state. Large FPs fragment are dominant in the autumn and winter. Small pristine eggs are dominant in the winter while in the summer large, degraded eggs are more abundant.

The present study illustrates the important role that *P. antarcticum* can play in driving the sinking flux of POC in the Southern Ocean, a region which contributes significantly to the global C export production^32^. We find that Antarctic silverfish in Terra Nova Bay account for 41% of annual POC flux (in term of eggs and faeces) and that the variability of this flux is modulated by spawning strategy. The schematic (Fig. 4) summarises the mean contribution for each season of fish egg and FP to the POC. As well as supporting the findings of other studies regarding the important contribution of fish FPs^13,14^ in different regions, this study further identifies the major contributions made by sinking eggs. In addition to being a major source of sequestration, both FP and eggs flux can also be a major driver of productivity in benthic communities, particularly in shelf regions which are their specie’s spawning area. Further, this study adds new insight in the Antarctic silverfish reproduction strategy by providing the first year-round observation of eggs flux.

Increasing water temperature in the Terra Nova Bay (and in general in the Southern Ocean), as a result of climate change, will likely have a negative impact on fish population^43^. The Antarctic silverfish have evolved a suite of specific ecological and physiological adaptations to the environmental conditions in the cold and highly seasonal Antarctic continental shelf^43,46^. Therefore, a sudden change in environmental conditions driven by the current rapid climate change could negatively affect this weak equilibrium. Previous studies have already shown declines in local populations of the Antarctic silverfish^47^. Our results suggest that a potential decrease in fish biomass is likely to impact not only the Antarctic food web but also the marine carbon budget. To connect climate variability, fish population dynamic and carbon cycle is then fundamental to increase confidence in forecasting the effect of climate change. Furthermore, the role of this organism in the biogeochemical cycle should be also taken into account (in addition to the role in the food web) to improve conservation measure and policy.

We are aware that other fish species might represent an unknown fraction of the samples found in the sediment trap. However, overall, our results highlight the relevance of pelagic fishes as an important biological vector of carbon export in this region. Further, we assume that the large majority of the observed eggs and faeces are likely being produced by Antarctic silverfish because of contrasting feeding/spawning behaviour of some of the most common fishes in this region (i.e. *C. hamatus, T. bernacchii* and *T. newnesi*)^42^ in combination with the position of the mooring (located in the nursery and hatching area of this organism). Future research focus on genetic analyses of eggs and faeces will be crucial to fully discriminate and quantify the individual carbon export contribution of key fish species in the Southern Ocean.

## Methods

### Study area

**Fig. 5:**
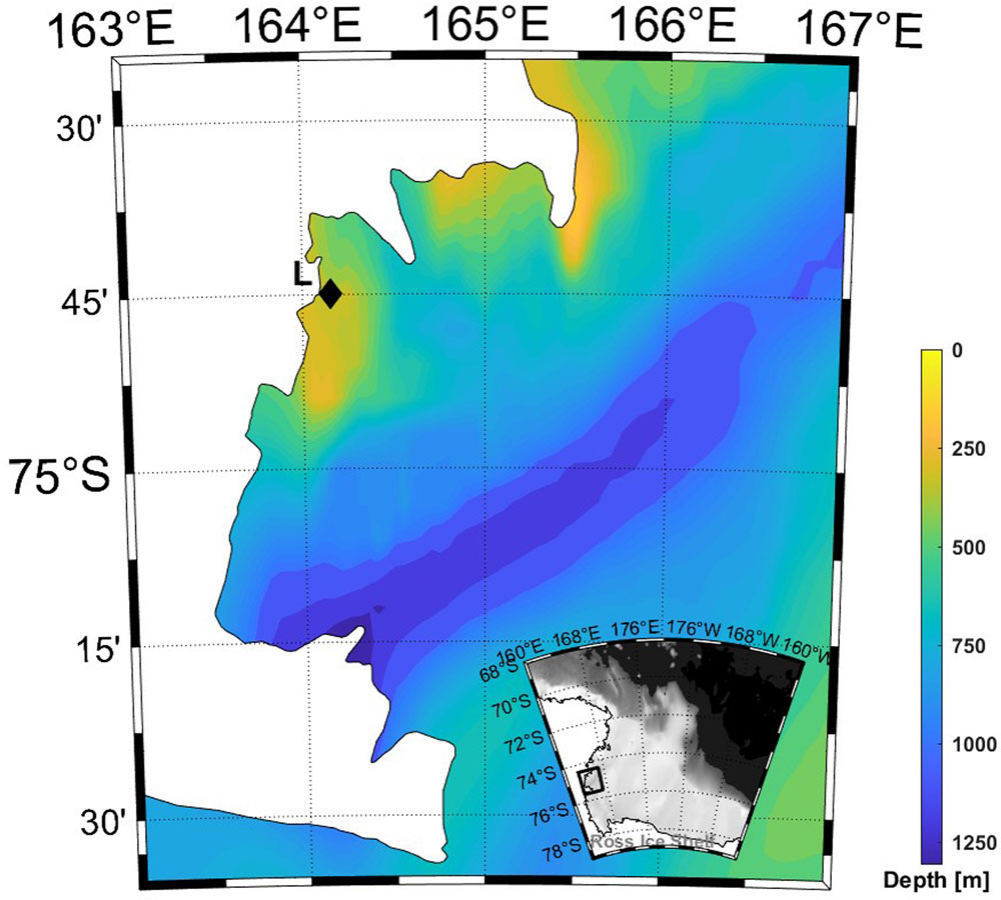
Location map. Location of sediment trap mooring (L) in the Terra Nova Bay, Western sector of Ross Sea, Southern Ocean. Map with bathymetry overlaid shown in colour (M_Map: A mapping package for MATLAB”, version 1.4 m). Source of bathymetry data Nitsche and Davey^48^. The black diamond indicates mooring L position.

TNB is located in the western sector of the Ross Sea, along the Victoria Land coast (Fig. 5). The most important feature of TNB is a persistent coastal latent heat polynya^48,49^. The Terra Nova Bay polynya acts as an ice factory during winter and has a primary function in the sea-ice local dynamics^50,51^. The strong katabatic winds blowing offshore form polynya as they advect the newly formed ice far from the coast^52^. As a result, a major environmental feature of TNB is the seasonal sea-ice cover, bordering coastal areas for almost 9 months of the year^53^. The polynya of TNB is known to play a crucial role in the formation of the High Salinity Shelf Water the densest water mass of the Southern Ocean, that directly contributes to the oceanic bottom water formation^54,55^.

### Sediment trap sampling

Sediment trap samples are collected by a Hydro-Bios Multi Sediment Trap MST 24, equipped with 24 receiving cups and a collecting area of 0.6 m^2^. The traps were deployed in TNB Polynya (mooring L: 74°44.6048′S, 164°8.3916′E, bottom 145 m) at 100 m depth during 1999. The sample carousel of the sediment trap is set up to rotate at intervals of 15 days over the whole year. Each trap is fitted with a plastic baffle mounted in the opening, to prevent the entrance of large-sized organisms. In the receiving cups, 4% buffered formalin-seawater solution is used as preservative. Upon recovery, samples are stored at ∼2–4 °C in the dark until further analysis.

The mooring line is equipped with an oceanographic instrument to monitoring currents (Aanderaa RCM7) and a conductivity/temperature/depth logger (CTD, SBE37 Microcat). Details of hydrographic conditions at the mooring site have been published in ref. ^56^.

### POC, FPs and eggs analysis

Each sample is split accurately into a series of pseudo-replicate fractions, for subsequent analysis, following the technique of ref. ^57^.

Prior to splitting, ‘swimmers’, i.e. zooplanktonic organisms that can enter the receiving cups while alive, are carefully removed. For this purpose, samples are first wet-sieved, through a 1 mm nylon mesh and the remaining swimmers are carefully removed by hand-picking under a dissecting microscope (dissecting microscope Olympus SZX16).

Replicate fractions are vacuum filtered through pre-weighed and pre-combusted (550 °C for 5 h) 25 mm glass fibre filters (GF/F, nominal pore size 0.7 μm). For POC determination, filters are pre-treated with 2 N H_3_PO_4_ and 1 N HCl. Filters are then desalted through briefly washing with distilled water and dried at 60 °C. POC were measured by combustion in an elemental analyser. CE Instruments NA2500 elemental analyser, was calibrated using an acetanilide calibration standard with a known % C of 71.09%. Standards are interspersed regularly between samples to measure and correct for drift. Analytical precision is better than 1.0%^34^.

Fish eggs and fish faeces are measured and counted using an ocular micrometre under light microscopy (dissecting microscope Olympus SZX16 and Leica M165 stereomicroscope). Fish eggs are identified according to ref. ^25^. For each sample 40 fish eggs and 40 fish faeces are randomly picked up for the calculation of carbon content by following the same protocol as for POC analyses.

POC, FP and egg fluxes are expressed in mg C m^−2^ d^−1^, estimated by dividing the total mass per sample by the time interval and the trap collection area. The carbon contribution is calculated as % of eggs and fish carbon to the total POC flux.

Dead eggs are identified as indicative of the natural mortality of eggs (i.e. caused by genetic abnormalities and incomplete fertilisation) and classified on the base of two stages of degradation (1-early decomposition, 2-physical decay) following the description of ref. ^58^.

#### Statistical analysis

Wilcoxon signed-rank test is used to determine whether there are any significant differences with regards to the different flux components within the sampling periods (in terms of variability in class size and carbon content of both FPs and eggs). Differences are considered significant where *p* < 0.05. Benjamini-Hochberg correction was applied for multiple comparisons. All the analyses were performed using the R software version 3.4.2.

### Reporting summary

Further information on research design is available in the Nature Portfolio Reporting Summary linked to this article.

### Supplementary information

Supplementary information

Reporting Summary

## Data Availability

All data are available at NERC EDS UK Polar Data Centre. 10.5285/49047818-D36A-45CE-8AD1-3D459E1CCEAD and presented in the Supplementary material.
