# Supplementary material for: Life strategy of Antarctic silverfish promote large carbon export in Terra Nova Bay, Ross Sea
Source: Commun Biol. 2024 Apr 11;7:450. doi: 10.1038/s42003-024-06122-8 (PMC11009349; doi:10.1038/s42003-024-06122-8)
Supplement: Supplementary file 1 — Supplementary information [file 42003_2024_6122_MOESM1_ESM.pdf]

Supplementary material

Life strategy of Antarctic Silver fish promote large carbon export in Terra Nova Bay, Southern Ocean

Manno et al.

	POC	FP	EG	SD POC	SD FP	SD EG
FEB	128.44	69.28	18.73	1.27	7.47	1.80
FEB	153.06	67.67	21.89	7.78	12.26	2.98
MAR	26.06	5.45	0.02	2.12	1.57	0.00
MAR	10.78	2.53	0.01	0.64	1.14	0.00
APR	10.06	3.61	0.03	1.81	1.03	0.00
APR	16.40	5.11	0.02	5.58	0.48	0.00
MAY	5.56	2.45	0.01	1.41	1.09	0.00
MAY	4.45	0.77	0.00	1.41	0.64	0.00
JUN	5.71	1.90	2.92	1.46	2.48	0.60
JUN	5.67	0.30	2.32	0.16	0.22	0.81
JUL	7.21	0.70	4.77	0.78	0.75	0.61
JUL	7.14	1.41	4.89	0.53	1.63	1.29
AUG	6.12	0.34	2.52	0.60	0.29	1.67
AUG	5.58	0.14	0.42	2.96	0.13	0.11
SEP	7.33	1.13	0.69	2.35	1.41	0.36
SEP	6.73	1.81	0.61	1.30	1.03	0.53
OCT	2.57	2.06	0.60	3.48	1.32	0.71
OCT	12.98	3.40	0.42	2.32	0.24	0.18
NOV	16.40	10.21	1.46	1.18	5.25	1.40

Supplementary Table 1 Data set of Particulate Organic carbon (POC) flux and the contribution to the POC flux by fish krill Faecal Pellets (FP) and fish eggs (EG). Data are expressed as mg C m^{-2} d^{-1}, at L mooring station, Terra Nova Bay Polynya, Ross Sea, during February 1998-November 1998.

	FP 2-4.5 mm	FP 4.5-7 mm	Eggs 1.8-2mm	Egg 2-2.5mm
FEB	60	40	48	54
FEB	67	33	46	50
MAR	74	26	51	52
MAR	45	55	0	0
APR	43	57	0	0
APR	35	65	0	0
MAY	44	56	0	0
MAY	33	67	0	0
JUN	44	56	69	31
JUN	46	54	67	34
JUL	34	66	71	29

**JUL**	44	56	64	32
**AUG**	41	59	69	32
**AUG**	56	44	72	30
**SEP**	34	66	42	58
**SEP**	56	44	45	55
**OCT**	65	35	44	61
**OCT**	76	24	40	54
**NOV**	75	25	46	55

Supplementary Table 2 Data set of fish FP and eggs class size (as %) of Antarctic Silver fish measured from eggs collected in the sediment trap samples deployed at L station, Terra Nova Bat, Ross Sea, during February 1998-November 1998. Note most of the fish FP are present as fragments.

	**Pristine**	**Stage 1**	**Stage 2**
**FEB**	0	0	100
**FEB**	0	0	100
**MAR**	NA	NA	NA
**MAR**	NA	NA	NA
**APR**	NA	NA	NA
**APR**	NA	NA	NA
**MAY**	NA	NA	NA
**MAY**	NA	NA	NA
**JUN**	76	20	4
**JUN**	78	19	3
**JUL**	76	21	3
**JUL**	78	18	4
**AUG**	79	18	3
**AUG**	66	32	2
**SEP**	65	33	2
**SEP**	52	41	7
**OCT**	49	43	8
**OCT**	47	47	6
**NOV**	46	45	9

Supplementary Table 3 Data set of egg degradation stage (as %) measured from eggs collected in the sediment trap samples deployed at L station, Terra Nova Bat, Ross Sea, during February 1998-November 1998. Pristine (no sign of degradation), stage 1 (early stage of decomposition), stage 2 (advanced stage of decomposition, i.e. physical decay). NA: no investigation because eggs were almost absent in this period.
